# Acid-Fast Bacilli Other than Mycobacteria in Tuberculosis Patients Receiving Directly Observed Therapy Short Course in Cross River State, Nigeria

**DOI:** 10.1155/2012/301056

**Published:** 2012-07-30

**Authors:** Benjamin Thumamo Pokam, Anne E. Asuquo

**Affiliations:** ^1^Department of Medical Laboratory Science, University of Buea, P.O. Box 63, Buea, Cameroon; ^2^Department of Medical Laboratory Science, University of Calabar, Calabar, Nigeria

## Abstract

The information on the contribution of non tuberculous mycobacteria (NTM) to mycobacterial infections in Africa is scarce due to limited laboratory culture for its isolation and identification. One hundred and thirty-seven sputum smear positive patients were recruited into a study on the molecular epidemiology of *Mycobacterium tuberculosis* in Cross River State. Following sputum culture, 97 pure isolates were obtained and identified using Capilia TB-Neo and further confirmed by the GenoType Mycobacterium CM kit. Of the 97 isolates, 81 (83.5%) isolates were Capilia TB-Neo positive while 16 (16.5%) were Capilia TB-Neo negative. Further confirmation with the GenoType Mycobacterium CM kit revealed that 4 (25%) of the 16 isolates belonged to NTM and included *M. fortuitum I, M. fortuitum II/M magaritense, M. abscessus,* and *M. avium* ssp. The remaining 12 (75%) Capilia TB-Neo negative isolates were not members of the genus *Mycobacterium* despite their AFB appearance. Six (33.3%) of the Capilia TB-Neo negative were from HIV positive tuberculosis patients. All subjects in this study were placed on DOTS shortly after the AFB results were obtained. The implication of isolation of 16.5% nontuberculous isolates further emphasizes the need for culture of sputum specimen especially in HIV positive patients prior to administration of antituberculosis therapy.

## 1. Introduction

*Mycobacterium tuberculosis* is the most important causative agent of tuberculosis (TB) while nontuberculous mycobacteria (NTM) may play a key role in etiology of TB-like syndromes [1].

Data on nontuberculous mycobacterial disease in sub-Saharan Africa are limited, due mainly to the lack of laboratory culture facilities for the identification of mycobacterial species. Consequently, many laboratories do not discriminate between *M. tuberculosis* and NTM for similar reasons [2–4]. Treatment of TB patients in most sub-Saharan African countries including Nigeria is based solely on the results of microscopic smear positivity. As such, all sputum smear positive diagnosed patients are indiscriminately placed on DOTS, the current international TB treatment strategy. The implication is that NTM is inappropriately managed with first-line antituberculous drug [4, 5], worsening the patient's condition and raising the risk of drug resistance. Although it is known that most sputum smear positive patients are truly TB patients [6], the continued increase in TB drug resistance raises the question on the impact of this indiscriminate use of TB drugs to treat all diagnosed sputum smear positive patients. In assessing the molecular epidemiology of *Mycobacterium tuberculosis* complex in the Cross River State of Nigeria, the data revealed the involvement of AFB other than mycobacteria in tuberculosis-like symptoms in the population.

## 2. Materials and Methods

### 2.1. Patients and Setting

The study was carried out in Cross River State, located in the South-South part of Nigeria. Patients included 137 smear positive patients from which 3 consecutive sputum specimens were collected. Patients were recruited over a period of 12 months (June 2008 to May 2009) from the major hospitals and TB care facilities in the north, central, and south senatorial districts of the state. Both genders and age groups of 10 to 70 years were included. Permission to carry out the work was obtained from the local ethical committee, and informed medical consent was equally obtained from all participating patients.

### 2.2. Sputum Cultures

Sputum specimen obtained from patients was preserved using sodium carbonate (75 mg) and/or refrigerated until cultured. Specimens were decontaminated using modified Petroff's method [7] and cultured using BACTEC 960 (Becton Dickinson, Franklin Lakes, NJ07417, USA). Smears were made from isolates obtained from the BACTEC MGIT tubes, stained by the Ziehl Neelsen staining method, and examined for the presence of acid-fast bacilli (AFB). The growth on AFB positive MGIT tubes was further inoculated into two Lowenstein-Jensen slants, one containing sodium pyruvate. The cultures were examined twice a week and their rate of growth and colonial morphologies recorded. Contaminated slants were further re-decontaminated and recultured. Negative cultures and discarded heavily contaminated slants reduced our working cultures to 97 pure isolates. 

### 2.3. Identification of Isolates

Primary identification of organisms using Capilia TB-Neo (TAUNS Laboratories, Inc. Japan), an immunochromatographic method which can detect MPT64, a protein specifically secreted by *M. tuberculosis* complex and not produced by NTM, was performed according to the manufacturer's instructions (http://capilia.jp/english/capilia_tb_neo.html). 

To determine the species of the Capilia TB-Neo negative isolates, the GenoType Mycobacterium CM kit, a test based on the DNA-STRIP technology that permits the identification of some mycobacterial species, was used according to the manufacturer's instructions (Hain Lifescience GmbH, Nehren, Germany). Briefly, DNA extraction was carried out by sonication on the cultured organisms, heat killed at 80°C for 30 minutes, followed by a multiplex amplification with biotinylated primers and a reverse hybridization. The hybridization included the chemical denaturation of the amplification products, hybridization of a single-stranded, biotin-labeled amplicons to membrane-bound probes, stringent washing, addition of a streptavidin/alkaline phosphatase conjugate, and an alkaline phosphatase mediated staining reaction. A template ensured the easy and fast interpretation of the banding pattern obtained. Three controls (conjugate, universal, and genus) are included in each strip.

## 3. Results

Following culture and identification of the 97 isolates in this study, 81 (83.5%) were identified as members of the* Mycobacterium tuberculosis *complex (data published [8]) using both immunochromatographic technique (Capilia TB-Neo positive) and GenoType Mycobacterium CM. Eighteen (18.6%) patients were HIV positive. However, 21 individuals did not know their HIV status at the time the study was carried out. Of the 18 HIV positive TB patients, 6 (33.3%) were Capilia negative (*P* = 0.09) (Table 1).

Of the 16 Capilia negative isolates, GenoType Mycobacterium CM molecular test identified 4 isolates as *M. fortuitum I, M. fortuitum II/M magaritense, M. abscessus, *and *M. avium *ssp. Two of the 4 isolates were obtained from HIV positive patients. Twelve AFB smear positive isolates were found not to be members of the genus mycobacteria, as shown in Table 2.

## 4. Discussion

In recent times, several newer immunochromatographic techniques have emerged (e.g., Capilia) making rapid differentiation between *M. tuberculosis* complex and NTM possible and less cumbersome, even though they cannot identify the NTM species. Although it has been shown that most cultures-positive mycobacteria are *M. tuberculosis* in regions where tuberculosis is highly prevalent [6, 9], NTM isolates have been increasing gradually. Up to 20–30% of AFB smear positive isolates have been identified as NTM in Korea [10]. These organisms can cause true infection and disease and can be important clinically [11]. In Africa, the contribution of NTM to such disease has been examined on a small scale only [1]. Two previous studies in Nigeria show that Fawcett and Watkins [12] did not isolate mycobacteria other than those causing tuberculosis in their study in the northern region of the country. However, 11% atypical Mycobacteria have been reported in Lagos in 1986 [6]. The latter authors classified six as *M. avium*, four as *M. kansasii,* and one as *M. fortuitum*. The four NTM species isolated in this study included *M. fortuitum* I, *M. fortuitum* II/*M. magaritense*, and *M. avium* species besides M. abscessus. Two studies carried in the country have equally isolated *M. fortuitum*, *M. intracellulare, M*.* chelonae, M. avium,* and *M. kansasi* among sputum smear positive samples/culture isolates [9, 13].

Most NTM disease cases involve species of *M. avium* complex, *M. abscessus, M. fortuitum, *and* M. kansasii*. *M. abscessus* is being seen with increasing frequency and is particularly difficult to treat medically [14], because the disease caused by *M. abscessus* often progresses slowly over years and because older adults are typically affected. In more developed countries, *M. avium* and *M. simiae* are responsible for disseminated disease in HIV-infected persons [15]. The HIV era has brought an increase of infection by these opportunistic human pathogens. Nontuberculous mycobacteria are involved in a range of diseases including pulmonary disease, hypersensitivity pneumonitis, cervical lymphadenitis, and disseminated infection. The disseminated infection is generally associated with HIV infection: AIDS and immunosuppression [16]. Though this study did not show any association between the NTM and HIV infection, this could be attributed to the number of patients that did not know their status at the time of specimen collection. In 2008, when patients in this study were being sampled, HIV counseling and testing in Nigeria were not compulsory for TB suspects or patients newly diagnosed with TB. Moreover, in 2007, only 3 percent of health facilities in Nigeria had HIV testing and counseling services [17]. 

Of the initial 16 NTM isolated in this study, molecular techniques showed that 12 were not members of the genus *Mycobacterium*, despite the fact that they were isolated from sputum smear positive patients. It may be that these organisms are *Nocardia* species or *Rhodococcus equi*, since their microscopic appearance was consistent with AFB. The incidence of nocardiosis especially the former *Nocardia asteroides* complex has been on the increase due to the increase in the number of immunosuppressed patients during recent decades [18]. More than 70% of patients with nocardial infection are immunocompromised, and disseminated nocardiosis is associated with several immunocompromising conditions. More recently, HIV infection has been described as a risk factor for disseminated nocardiosis [19]. Pulmonary nocardiosis is very often misdiagnosed as tuberculosis and treated with antitubercular drugs. Such cases may end fatally [20].

## 5. Conclusion

The challenge therefore in most African countries, and especially in Nigeria, still remains the introduction in a large scale of laboratory culture for the specific identification of the mycobacteria. The data obtained in this study provide some evidence of the role of nontuberculous AFB organisms and the public health implications of DOTS administration without sputum culture. Sputum culture should be performed in smear positive patients with known HIV positive status or HIV patients with clinical symptoms of tuberculosis, so as to identify the strains involved and thus avoid unnecessary administration of anti-TB drugs with risk of drug toxicity for the patients and increase drug resistance. Half (2/4) of the patients in this study with the identified NTM were HIV positive. Moreover, drug resistance should not be systematically considered as it is currently the case, especially in patients still AFB smear positive following intensive phase treatment in settings devoid of culture facilities, as such patients may be either infected by NTM or AFB not members of the genus mycobacteria.

## Figures and Tables

**Table 1:** Association of mycobacteria genus and HIV status of subjects.

HIV status		Capilia TB-Neo	
	Positive (%)	Negative (%)	Total
Positive	12 (66.7)	6 (33.3)	18
Negative	50 (86.2)	8 (13.8)	58
Unknown	19 (90.5)	2 (9.5)	21

Total	81	16	97

**Table 2:** Genotype mycobacteria identification of Capilia TB-Neo negative isolates from sputum smear positive patients.

Isolates no.	Sex/Age	Strains	HIV status
0015IDH	F/25–34	NM	NEG
0033TH	F/25–34	NM	POS
0043TH	M/35–44	NM	NEG
0053TH	M/35–44	NM	POS
0073IDH	M/45–54	*M. fortuitum *I	POS
0082IDH	M/25–34	NM	UNK
0083IDH	F/25–34	NM	NEG
0086IDH	F/25–34	NM	POS
0089IDH	M/25–34	*M. fortuitum* II	NEG
		/*M. magaritense *	
0094IDH	F/25–34	NM	NEG
0105TH	F/15–24	NM	NEG
00111IDH	M/25–34	NM	POS
00113IDH	M/15–24	NM	NEG
00128TH	M/25–34	NM	NEG
00134TH	M/35–44	*M. abcessus*	POS
00407OG	F/35–44	*M. avium* ssp.	UNK

F: female; M: male; NM: not member of the genus mycobacteria; POS: positive; NEG: negative; UNK: unknown.
